# Evaluating drill speed effects on tunnel diameter in ACL reconstruction: Insights from an experimental animal study

**DOI:** 10.1002/jeo2.70406

**Published:** 2025-09-24

**Authors:** Jamil Alasov, Nelo João Zeca Chihal Lima, Mehmet Erduran

**Affiliations:** ^1^ Department of Orthopedic Surgery and Traumatology Dokuz Eylül University Balçova İzmir Turkey

**Keywords:** anterior cruciate ligament, drill speed, rabbit, thermal necrosis, tunnel enlargement

## Abstract

**Purpose:**

To determine how drilling speeds affect tunnel enlargement during cruciate ligament reconstruction.

**Methods:**

Twelve adult female New Zealand rabbits, each weighing between 3 and 3.5 kg, were randomly assigned to two groups based on drilling speeds (400 RPM and 1500 RPM). Following sedation, the knee and tibia regions were shaved, and the knee joint was accessed using a medial parapatellar approach. The anterior cruciate ligament (ACL) was incised using a scalpel. Tibial and femoral tunnels were drilled with a 2 mm blunt‐tip drill at the assigned group speeds. Room temperature saline was used for irrigation throughout the drilling procedure. An extensor tendon graft was harvested from the anterior cruris and sutured using the Krakow technique with 4‐0 Vicryl. The graft was passed through the tunnel and secured to a pre‐placed screw near the tunnels. Post‐surgery, the operated extremity was not immobilised, and the rabbits were observed while moving freely within the cage. Twelve weeks post‐operatively, the animals were humanely euthanized, and micro‐computed tomography (CT) was employed to assess the diameters of the femoral and tibial tunnels at the proximal, middle and distal regions.

**Results:**

No statistically significant differences were found between radiological femoral and tibial tunnel diameter measurements of the 400 and 1500 RPM drilling groups 12 weeks after arthroscopically assisted ACL reconstruction in New Zealand rabbits. However, within the 400 RPM group, there was a measurable increase in the tunnel diameter in the proximal tibia and distal femur regions.

**Conclusion:**

The findings of our study suggest that concerns about higher drilling speeds in ACL surgery may be overstated, as higher speeds did not lead to significant radiological tunnel enlargement.

**Level of Evidence:**

N/A.

**Abbreviations**
ACL: anterior cruciate ligament
Micro‐CT: micro‐computed tomography
RPM: revolutions per minute
°C: degrees centigrade

## INTRODUCTION

Anterior cruciate ligament (ACL) injuries are the most prevalent form of knee ligament damage and are increasingly common due to rising athletic participation. This trend has resulted in 130,000–200,000 ACL reconstruction procedures annually in the United States and about 400,000 globally [19]. ACL reconstruction is generally effective in restoring knee stability and allowing individuals to return to their pre‐injury activity levels. However, 0.7%–20% of patients experience failure [11, 25]. ACL procedures' total cumulative failure rate ranges from 3.2% to 27%, averaging 11.9%. At long‐term follow‐up, at least one in nine patients undergoing ACL reconstruction will experience re‐rupture or clinical failure [9]. Failure after ACL reconstruction can vary based on several factors, including tunnel misplacement, graft re‐re‐rupture, and tunnel enlargement [9, 11, 25]. Tunnel enlargement is particularly concerning as it can lead to catastrophic failure of the graft [38]. Enlargement typically occurs within the first 3 months post‐surgery, can continue for up to 2 years, and stabilises [13].

Tunnel enlargement following ACL reconstruction has been a significant research focus, and various causes and theories have been proposed; however, the precise aetiology of tunnel enlargement remains uncertain, with studies pointing to mechanical factors (such as graft‐tunnel micromovements, bungee effect, the windshield‐wiper effect, accelerated rehabilitation, higher initial graft tension and improper graft placement) and biological factors (such as cytokine activity and heat‐induced necrosis) contributing to the phenomenon [13, 15, 18, 21, 33, 37]. Upon reviewing the literature, we realised that some studies have been conducted with drills ranging from manual to motorised ones operating at speeds up to 100,000 RPM, involving in vitro and in vivo animal experiments. These studies have been analysed radiologically and histologically [2, 20, 30]. The rotational action of the drilling tip during tunnel creation produces heat, which can lead to bone necrosis if temperatures exceed critical levels [2, 35]. Several studies indicate a linear relationship between drill speed and temperature increase up to 10,000 revolutions per minute (rpm). Bone tissue is especially vulnerable to temperatures over 47°C. In 1980, Lavelle and Wedgwood found that temperatures could reach 60°C even with irrigation, and subsequent studies have observed temperatures climbing as high as 100°C [22]. This thermal damage can compromise bone healing, hinder osseointegration, and ultimately result in implant instability or failure. Despite awareness of these thermal effects, the impact of drill speed on surgical outcomes has not been extensively studied.

Using radiological methods, we aimed to examine the effect of necrosis caused by drill rotational speeds on tunnel enlargement in tunnels drilled at speeds of 400 RPM and 1500 RPM. Using speeds akin to those in human ACL reconstruction, we hypothesized that elevated drilling speeds, particularly around 1500 rpm, increase the risk of thermal necrosis and cause tunnel enlargement. Drills with a sharpening angle identical to those in clinical practice were used. The choice was partly guided by suitability for the animal model used.

## MATERIALS AND METHODS

Initially, we conducted a literature review of similar animal studies, which revealed a gap in existing data.

**Figure 1:**
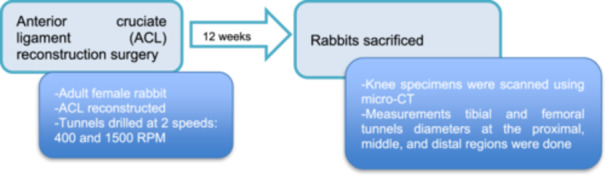
Flow chart of the study design.

During the study design (see Figure 1), the total number of animals included (sample size) was determined using the resource equation method [3, 7]. We then secured approval from the Local Ethics Committee (Protocol No. 23/2021) and financial support from the Scientific Research Projects (BAP). Following this, we procured the experimental animals. The experiments were conducted at the Dokuz Eylül University Faculty of Medicine Multidisciplinary Experimental Animals Laboratory. Radiological analyses were performed at the Central Research Testing and Analysis Laboratory Application and Research Center (EGE MATAL Micro CT Lab). All procedures adhered to the Guide for the Care and Use of Laboratory Animals and received approval from our Institutional Animal Care and Use Committee.

### Animal preparation

Thirteen adult female New Zealand rabbits weighing 2.5–3 kg and exhibiting regular activity were procured for the study. These rabbits were housed in our university's experimental animal laboratory under standard laboratory conditions (room temperature of 20°C–24°C, 50% humidity) and were fed ad libitum with free access to food and water. One rabbit was initially designated for a pilot experiment to ensure the feasibility of the surgical procedures, as detailed in the ‘Surgery’ section. The remaining 12 rabbits were randomly assigned to two different experimental groups of six (*n* = 6): one with a drill speed of 400 revolutions per minute (RPM) and the other at 1500 RPM. This division aimed to evaluate the effects of different drilling speeds on tunnel enlargement.

### Surgery

Sedation‐analgesia was administered intramuscularly to all subjects using 35 mg/kg ketamine and 5 mg/kg xylazine. Following drug administration, a sedation onset occurred within an average of five minutes. Cefazolin was administered intramuscularly for surgical infection prophylaxis at a dose of 50 mg/kg. Throughout anaesthesia, vital signs were continuously monitored to ensure stability. After sedation, rabbits were immobilised in a supine position. Each subject's randomly selected knee and leg were shaved, disinfected with polyvidone iodine (Batticon), and sterilely draped. An incision approximately 5 cm in length was made on the anterior of the right knee, and a 3 cm incision was made on the anterior tibia.

**Figure 2:**
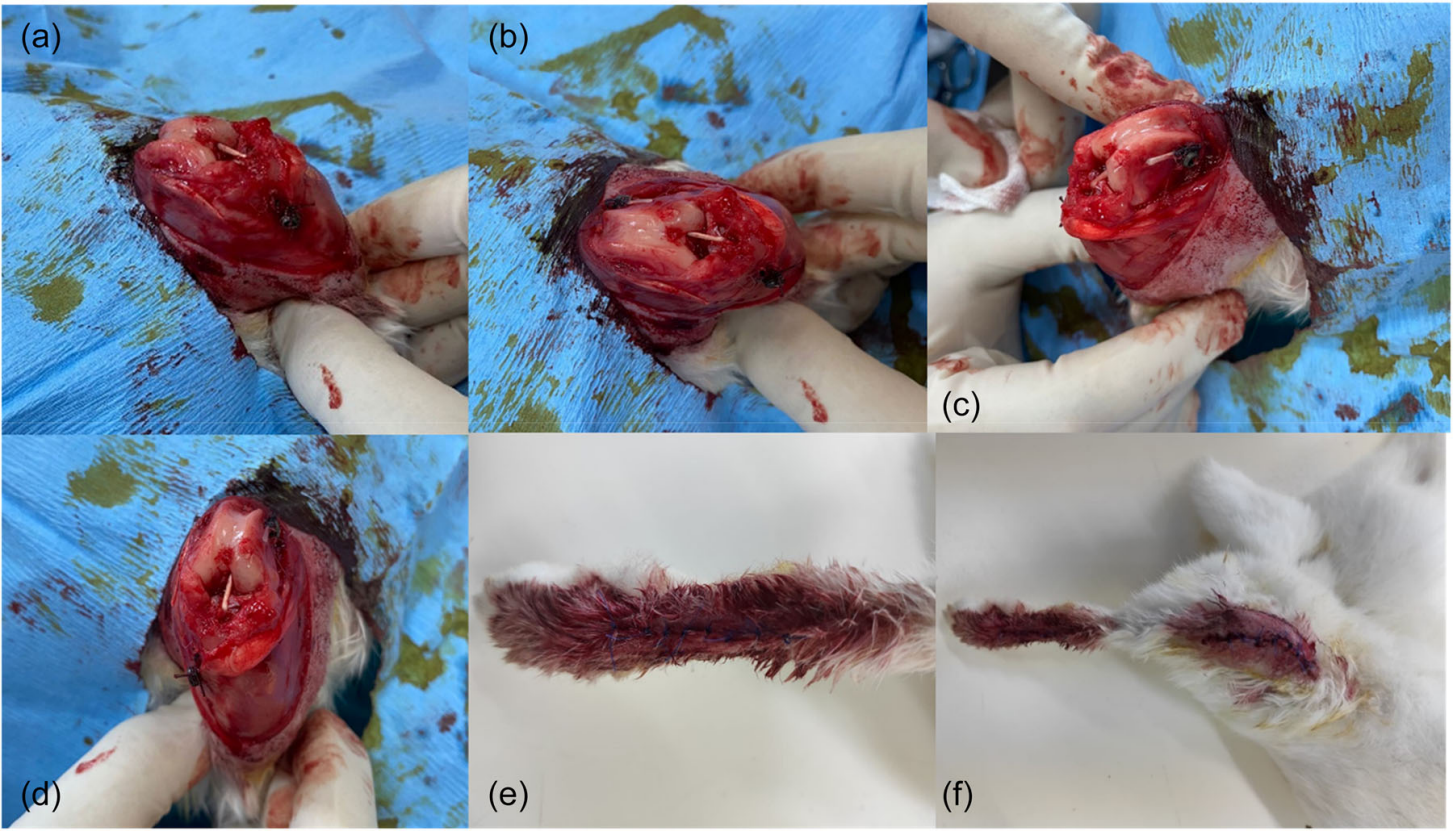
(a–d) Intraoperative views of the left knee of the experimental animal from the pilot study, captured from various angles. (e, f) Views of the wound sites following the surgical procedure.

The joint was accessed using a medial parapatellar approach, and the patella laterally deviated to expose the anterior cruciate ligament (ACL). For ACL transection, the knee was flexed, and the ligament was severed with a scalpel. Tibial and femoral tunnels were created at the ACL footprint using a 2.0 mm blunt‐tipped drill. The drill speed was set to 400 RPM for Group 1 and 1500 RPM for Group 2. During the drilling process, irrigation with a saline solution was performed to replicate the environment in which drilling was done in human subjects during ACL reconstruction. An extensor tendon graft was harvested from the anterior cruris, and both ends of the graft were sutured using the Krakow suture technique with 4‐0 Vicryl. A screw measuring 10 mm in size was placed 5 mm distal to the tibial tunnel and 5 mm proximal to the femoral tunnel. After passing the prepared graft through the tunnels, the sutures were tied to the screw with the knee in a mid‐flexion position. The graft's integrity was checked by moving the knee into full flexion and extension positions. Post‐procedure, the wound site was irrigated with saline solution. The patella was reduced, and the medial parapatellar incision was closed with 4‐0 Vicryl. The skin was closed appropriately with 3‐0 Prolene. (see Figure 2 for visual reference on the procedure stages).

After the surgical procedure, the wound site was carefully cleaned with povidone‐iodine. For the first two days post‐surgery, paracetamol was administered orally to the subjects by adding 2 mg/kg to their drinking water for analgesia. No splint or immobilisation was applied to the subjects, and they were allowed to move freely in their cages. Wounds were treated daily with polyvidone iodine and were closely monitored for local or systemic signs of infection until they healed.

### Radiological evaluation

**Figure 3:**
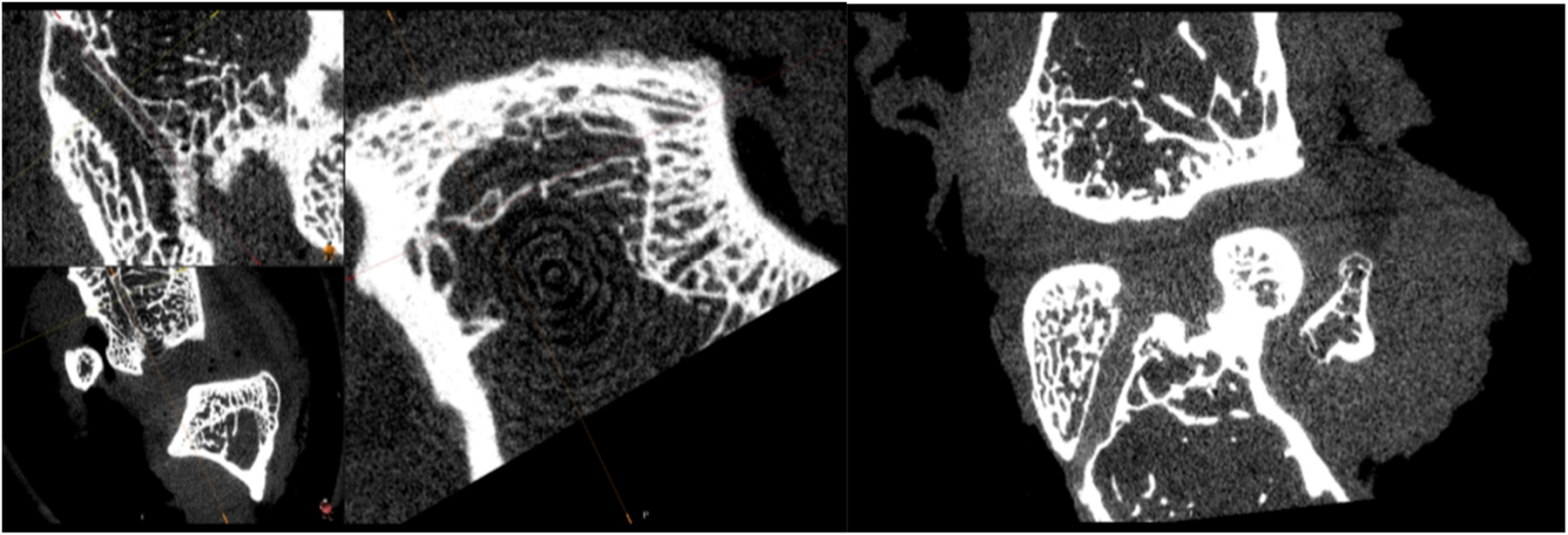
Sections of the computed tomography (CT) image measuring tunnel widening. The tunnel diameter was measured on the sagittal view in regions of interest (proximal, middle and distal) in the femoral and tibial tunnels.

At the end of the 12‐week follow‐up, all subjects were sacrificed using a high dose of sodium thiopental (Pental). After sacrifice, the operated knees were removed as a block, cutting from the proximal end of the screw fixed in the femur and the distal end of the screw in the tibia. The excised tissues were placed in 10% neutral‐buffered formalin. The specimens (*n* = 6 from each group at each time point) were scanned using a micro‐CT device with 30 μm resolution, and data was stored in DICOM format at the EGE MATAL Micro CT Laboratory. The obtained data were recorded and uploaded to the Dokuz Eylül University Department of Radiology. A total of six measurements were made in the entry, middle, and exit regions of the femoral and tibial tunnels using SECTRA IDS7 (Figure 3).

### Statistical analysis

The Statistical Package for Social Sciences (SPSS), version 24.0, was used to analyse the tunnel enlargement in the experimental animals of the two groups (400 RPM and 1500 RPM) relative to the 2 mm drill diameter, differences in tunnel diameters between groups, and differences within each group. Differences between the two group variables were analysed using an unpaired Student's t‐test, analysis of variance, and a Mann–Whitney *U* test as appropriate. The Shapiro–Wilk test was conducted to assess the normality of distribution for the group measurements, and no significant difference was found, indicating that the measurements followed a normal distribution (*p* < 0.05) (Table 1). For the Shapiro‐Wilk test and t‐test, a significance threshold value of *p* < 0.05 was set.

**Table 1:** A comparative analysis of radiological measurements was conducted on the distal, middle and proximal regions of the tibia and femur tunnels using a 2 mm diameter drill at 400 RPM and 1500 RPM groups.

One‐sample statistics						
		*N*	Mean	Std. deviation	Std. error mean	*p*‐value
400 RPM						
Tibia	Distal	6	0.683	0.3545	0.1447	0.000
	Middle	6	1.033	0.3983	0.1626	0.002
	Proximal	6	1.417	0.6524	0.2664	0.080
Femur	Distal	6	1.467	0.5888	0.2404	0.077
	Middle	6	1.233	0.4967	0.2028	0.013
	Proximal	6	1.117	0.4119	0.1682	0.003
1500 RPM						
Tibia	Distal	6	1.050	0.4135	0.1688	0.002
	Middle	6	0.967	0.3670	0.1498	0.001
	Proximal	6	1.067	0.5502	0.2246	0.009
Femur	Distal	6	1.550	0.3209	0.1310	0.019
	Middle	6	1.167	0.6501	0.2654	0.026
	Proximal	6	1.083	0.5492	0.2242	0.009

## RESULTS

Levene's test confirmed the homogeneity of variances across the groups. Since the measurements adhered to a normal distribution and established variance homogeneity, parametric tests were appropriate for analysis. Intergroup comparisons of radiological measurements were conducted using the t‐test, which revealed no statistically significant differences between the groups (*p* < 0.05).

The radiological measurements in the 400 rpm group with a 2 mm diameter drill found that a significant tunnel enlargement was observed in the proximal tibia and distal femur regions (*p* < 0.05), with no notable changes in the other areas (*p* < 0.05). Conversely, the 1500 rpm group showed no significant tunnel enlargement but a significant narrowing across all tunnel regions (*p* < 0.05) (refer to Table 1 for detailed results).

### Comparison within the groups

The radiological measurements in within‐group matches in the 400 RPM group showed no significant tunnel enlargement, but significant narrowing occurred in the distal tibia‐proximal femur region (*p* < 0.05). In the 1500 RPM group, no significant differences in tunnel enlargement were observed (*p* > 0.05) (refer to Table 2 for detailed results).

**Table 2:** Comparison of within‐group matches in the 400 and 1500 RPM groups.

Paired samples statistics						
		Mean	N	Std. deviation	Std. error mean	*p*‐value
400 RPM						
Pair 1	Tibia distal	o.683	6	0.3545	0.1447	
	Femur proximal	1.117	6	0.4119	0.1682	0.041
Pair 2	Tibia medial	1.033	6	0.3983	0.1626	
	Femur medial	1.233	6	0.4967	0.2028	0.340
Pair 3	Tibia proximal	1.417	6	0.6524	0.2664	
	Femur distal	1.467	6	0.5888	0.2404	0.849
1500 RPM						
Pair 1	Tibia distal	1.050	6	0.4135	0.1688	
	Femur proximal	1.083	6	0.5492	0.2242	0.454
Pair 2	Tibia medial	0.967	6	0.3670	0.1498	
	Femur medial	1.167	6	0.6501	0.2654	0.224
Pair 3	Tibia proximal	1.067	6	0.5502	0.2246	
	Femur distal	1.550	6	0.3209	0.1310	0.915

## DISCUSSION

Our study observed significant tunnel enlargement in the 400 RPM group, particularly in the proximal tibia and distal femur regions, when using a 2 mm diameter drill. This enlargement was attributed to the absence of postoperative knee fixation and resulting micro‐movements within the tunnel during early recovery. Conversely, the 1500 RPM group exhibited no tunnel enlargement, challenging the assumption that higher drill speeds lead to radiologically observable tunnel enlargement. Understanding the causes of radiologically observable tunnel enlargement after ACL reconstruction surgery remains complex and poorly understood. Researchers categorise these aetiological factors into mechanical and biological groups [38]. Among the biological contributors, drilling‐induced cell necrosis is particularly noteworthy. It is well‐established that bone tissue necrosis can occur at temperatures above 47°C for at least one minute or 43°C for 1 h [34]. Studies consistently demonstrate a linear relationship between drill speed and temperature increase up to 10,000 RPM, beyond which the relationship becomes inversely proportional [1, 2, 20]. For instance, Goran Augustin and others have found that temperature rises with drill speeds up to 1000 rpm, decreases over 10,000 rpm, and stabilises between 40,000 and 50,000 rpm [5]. Similarly, Reingewirtz et al. observed a direct correlation between drill speed and temperature from 400 to 7,000 rpm, which inverted from 7000 to 24,000 rpm and plateaued at 40,000 rpm [29]. These findings suggest complex interactions that require further exploration. Additional studies report bone tissue temperatures of 53.7°C, 50.2°C and 47.0°C at drill speeds of 900, 1050 and 1200 rpm, respectively [8]. Furthermore, Ender Ugutmen et al. identified early tibial and femoral tunnel enlargement, attributed to a non‐specific inflammatory reaction driven by the pendulum motion of the drill bit and thermal necrosis [36]. These findings align with previous research [17, 23, 27].

Many studies have shown a linear proportional relationship between drill speeds up to 10,000 RPM and temperature. However, studies at speeds over 10,000 RPM have shown an inverse relationship [14, 31]. Li Yue et al., in a 2020 article published in the Journal of Bone and Joint Surgery, suggested that bone necrosis caused by drilling significantly contributes to bone tunnel enlargement due to the generated thermal energy. This process may be further amplified by a secondary inflammatory response [39]. Fahey and Indelicato also noted a necrotic rim of resorbed cancellous bone around drilled areas, frequently cited as a potential cause for bone tunnel widening [12]. Studies consistently show thermal necrosis leads to bone resorption around implants, resulting in potential implant loosening and osteosynthetic failure [10, 32]. Several studies recommend cooling through irrigation to mitigate these effects, effectively reducing temperature during drilling [4, 28]. Cooling can be applied internally or externally, with Haider et al. and Benington et al. finding no significant advantage of one method over the other [6, 16]. In our study, we used saline solution irrigation during tunnel drilling to replicate the conditions of human ACL reconstruction and minimise thermal damage. Although we cannot definitively claim that cooling impacted tunnel enlargement, we theorise that it effectively mitigated thermal necrosis, particularly at 1500 RPM. This suggests that drill speed rather than thermal factors primarily influenced any observed effects on the bone. Matthews and Hirsch demonstrated that an external irrigation rate of 500 mL/min is particularly effective in keeping the bone temperature below the critical threshold of 47°C [24]. Additionally, research by Rupesh Kumar Pandey et al. highlights that factors such as drill speed, feed rate, and cooling system characteristics play crucial roles in controlling temperature increases during drilling [26].

### Limitations

While the study focused on establishing foundational insights into the interaction between drilling speed and tunnel dimensions, we recognise that temperature increases during drilling and associated factors, drilled material properties (hardness, rigidity and porosity), and the drill sharpening angle are vital components that could influence outcomes. The difference in the modulus of elasticity between rabbit spongy bone (80–150 MPa) and human spongy bone (350–650 MPa) is significant and affects the extrapolation of findings to humans. Future studies should consider using synthetic bone models with properties like human bone or larger animal models to better align with human biomechanics and explore a broader range of mechanical properties. By using irrigation during drilling, we intended to closely replicate the conditions of human ACL surgery, where irrigation is routinely employed to manage bone temperature. However, it could also have acted as a confounder by potentially masking or mitigating the thermal effects associated with different drilling speeds. Therefore, a control group without irrigation might have helped isolate the impact of irrigation from other variables. Furthermore, micro‐movement due to postoperative fixation failure, unmeasured graft diameter, possible joint fluid tunnel enlargement effects, unmeasured drill progression speed, and time spent in the tunnel during drilling can be acknowledged as a limitation of this study.

Despite these limitations, using rabbits provided a practical, cost‐effective model to control variables, and employing drills with a sharpening angle that matched those used in clinical settings allowed focus on drilling speed and bone response. While caution is needed when extrapolating to humans, this study lays the groundwork for more intricate studies focusing mainly on temperature rise during drills and incorporating additional factors like drill design, different cooling agents, and material properties.

## CONCLUSIONS

No statistically significant differences were found between radiological femoral and tibial tunnel diameter measurements of the 400 and 1500 RPM drilling groups 12 weeks after arthroscopically assisted ACL reconstruction in New Zealand rabbits. However, within the 400 RPM group, there was a measurable increase in the tunnel diameter in the proximal tibia and distal femur regions. This suggests that the commonly held assumptions regarding the risk of thermal necrosis from increased drilling speeds may be overstated, as higher speeds did not lead to significant radiological tunnel enlargement.

## AUTHOR CONTRIBUTIONS

**Jamil Alasov**: Literature review; surgery; data gathering; literature discussion. **Nelo Joao Zeca Chihal Lima**: Literature review; literature discussion; and writing–original draft. **Mehmet Erduran**: Conceptualisation; supervision; literature review.

## CONFLICT OF INTEREST STATEMENT

The authors declare no conflict of interest.

## ETHICS STATEMENT

All procedures in this study adhered to the Guide for the Care and Use of Laboratory Animals and were approved by the Institutional Animal Care and Use Committee at Dokuz Eylül University Hospital's Institutional Board review (approval number 23/202100).

## Data Availability

The data that support the findings of this study are available from the corresponding author upon reasonable request.
